# Tetra­kis­(μ-3-meth­oxy­benzoato-κ^2^ *O* ^1^:*O* ^1′^)bis[acetonitrilecopper(II)]

**DOI:** 10.1107/S1600536811012074

**Published:** 2011-04-07

**Authors:** Sanjib Kar, Antara Garai, Sukhen Bala, Chandra Shekhar Purohit

**Affiliations:** a School of Chemical Sciences, National Institute of Science Education and Research, Institute of Physics Campus, PO: Sainik School, Bhubaneswar, Orissa 751 005, India

## Abstract

The centrosymmetric binuclear Cu^II^ title complex, [Cu_2_(C_8_H_7_O_3_)_4_(CH_3_CN)_2_], has a paddle-wheel-type structure [Cu—Cu distance = 2.6433 (3) Å]. Each Cu^II^ ion is coordin­ated by four O atoms from two 3-meth­oxy­benzoate ligands and one acetonitrile N atom in a square-pyramidal geometry.

## Related literature

For applications of binuclear copper(II) complexes bridged by four benzoic acid groups in a paddle-wheel arrangement in inorganic synthesis, catalysis, magnetism and medicinal chemistry, see: Ozarowski (2008 ▶); Kirchner & Fernando (1980 ▶); Inoue *et al.* (1968 ▶); Bergant *et al.* (1994 ▶). For crystal structures of similar complexes, see: Lah *et al.* (2001 ▶). For the preparation of similar complexes, see: Bernard *et al.* (1989 ▶). 
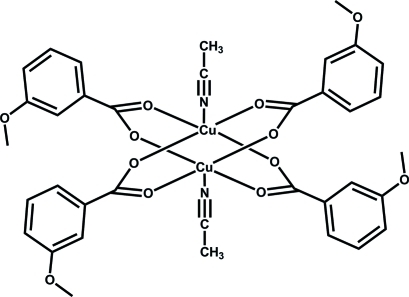


## Experimental

### 

#### Crystal data

[Cu_2_(C_8_H_7_O_3_)_4_(C_2_H_3_N)_2_]
*M* *_r_* = 813.73
Monoclinic, 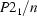

*a* = 7.2117 (7) Å
*b* = 19.6502 (3) Å
*c* = 12.6186 (9) Å
β = 90.016 (9)°
*V* = 1788.2 (3) Å^3^
*Z* = 2
Mo *K*α radiation
μ = 1.26 mm^−1^
*T* = 293 K
0.25 × 0.22 × 0.08 mm

#### Data collection

Bruker SMART CCD area-detector diffractometer
Absorption correction: multi-scan (*SADABS*; Bruker, 2007 ▶) *T*_min_ = 0.620, *T*_max_ = 0.746
43230 measured reflections
6872 independent reflections
4883 reflections with *I* > 2σ(*I*)
*R*_int_ = 0.036

#### Refinement

*R*[*F* ^2^ > 2σ(*F* ^2^)] = 0.034
*wR*(*F* ^2^) = 0.101
*S* = 1.01
6872 reflections
238 parameters
H-atom parameters constrained
Δρ_max_ = 0.40 e Å^−3^
Δρ_min_ = −0.34 e Å^−3^

### 

Data collection: *SMART* (Bruker, 2007 ▶); cell refinement: *SAINT* (Bruker, 2007 ▶); data reduction: *SAINT*; program(s) used to solve structure: *SIR92* (Altomare, *et al.* 1993 ▶); program(s) used to refine structure: *SHELXL97* (Sheldrick, 2008 ▶); molecular graphics: *XP* in *SHELXTL* (Sheldrick, 2008 ▶) and *DIAMOND* (Brandenburg, 1999 ▶); software used to prepare material for publication: *publCIF* (Westrip, 2010 ▶).

## Supplementary Material

Crystal structure: contains datablocks global, I. DOI: 10.1107/S1600536811012074/pk2314sup1.cif

Structure factors: contains datablocks I. DOI: 10.1107/S1600536811012074/pk2314Isup2.hkl

Additional supplementary materials:  crystallographic information; 3D view; checkCIF report

## Figures and Tables

**Fig. 1.**
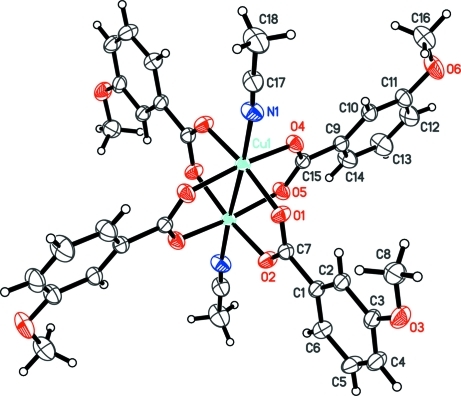
The molecular structure of the title compound. Displacement ellipsoids are drawn at the 30% probability level. H atoms are shown as small arbitrary spheres. Unlabelled atoms are related to their labelled counterparts by inversion [(x,y,z) to (2-x,2-y,-z)].

**Fig. 2.**
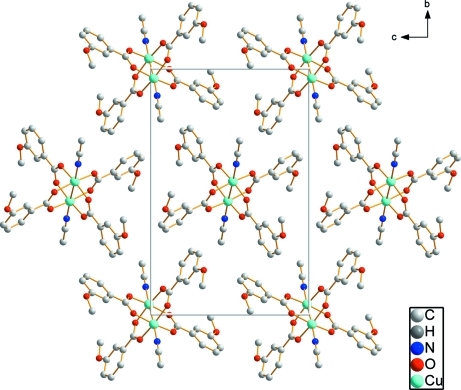
A packing diagram viewed along the crystallographic a axis.

## supplementary crystallographic information

### Comment

Binuclear copper(II) complexes bridged by four benzoic acid groups in a paddle-wheel arrangement are very important from the perspectives of molecular magnetism (Inoue, *et al.*, 1968).  These materials have potential applications in inorganic synthesis, catalysis, magnetism, and in medicinal chemistry (Ozarowski, 2008; Kirchner & Fernando, 1980; Inoue *et al.*, 1968; Bergant *et al.*, 1994).  Herein we report the structure of the title compound (Fig.1).  The structure is a centrosymmetric dinuclear Cu(II) complex having square pyramidal arrangement around each copper ion.  There is a lot of research interest for studying the effect of apical ligand (here the apical ligand is acetonitrile) in tuning the intramolecular magnetic exchange interaction between two Cu(II) ions in various paddle-wheel type dinuclear copper(II) benzoate complexes.

### Experimental

The title compound was obtained by addition of the solution of copper(II) acetate monohydrate (0.4 g, 2.0 mmol) in a 1:1 mixture of CH_3_CN + CH_3_OH (20 ml) to 3-methoxybenzoic acid (1.21 g, 8 mmol), and the mixture was stirred for 2 h. The green precipitate was filtered and washed thoroughly with diethyl ether (1.2 g, 80%).  Recrystallization from acetonitrile gave green crystals suitable for X-ray diffraction.

### Refinement

All H atoms were placed geometrically at idealized positions with C–H = 0.93 Å (C_ar_H) and 0.96 Å (RCH_3_), and were refined isotropically using a riding-model with *U*_iso_(H) = 1.2*U*_eq_ or 1.5*U*_eq_ (CH_3_ only).

### Crystal data

**Table:**

[Cu_2_(C_8_H_7_O_3_)_4_(C_2_H_3_N)_2_]	*F*(000) = 836
*M**_r_* = 813.73	*D*_x_ = 1.512 Mg m^−^^3^
Monoclinic, *P*2_1_/*n*	Mo *K*α radiation, λ = 0.71073 Å
Hall symbol: -P 2yn	Cell parameters from 9972 reflections
*a* = 7.2117 (7) Å	θ = 2.6–27.4°
*b* = 19.6502 (3) Å	µ = 1.26 mm^−^^1^
*c* = 12.6186 (9) Å	*T* = 293 K
β = 90.016 (9)°	Block, green
*V* = 1788.2 (3) Å^3^	0.25 × 0.22 × 0.08 mm
*Z* = 2	

### Data collection

**Table:**

Bruker SMART CCD area-detector diffractometer	6872 independent reflections
Radiation source: fine-focus sealed tube	4883 reflections with *I* > 2σ(*I*)
graphite	*R*_int_ = 0.036
φ and ω scans	θ_max_ = 33.5°, θ_min_ = 1.9°
Absorption correction: multi-scan (*SADABS*; Bruker, 2007)	*h* = −11→10
*T*_min_ = 0.620, *T*_max_ = 0.746	*k* = −30→29
43230 measured reflections	*l* = −18→19

### Refinement

**Table:**

Refinement on *F*^2^	Primary atom site location: structure-invariant direct methods
Least-squares matrix: full	Secondary atom site location: difference Fourier map
*R*[*F*^2^ > 2σ(*F*^2^)] = 0.034	Hydrogen site location: inferred from neighbouring sites
*wR*(*F*^2^) = 0.101	H-atom parameters constrained
*S* = 1.01	*w* = 1/[σ^2^(*F*_o_^2^) + (0.0531*P*)^2^ + 0.2391*P*] where *P* = (*F*_o_^2^ + 2*F*_c_^2^)/3
6872 reflections	(Δ/σ)_max_ = 0.001
238 parameters	Δρ_max_ = 0.40 e Å^−^^3^
0 restraints	Δρ_min_ = −0.34 e Å^−^^3^

### Special details

**Table:**

Geometry. All e.s.d.'s (except the e.s.d. in the dihedral angle between two l.s. planes) are estimated using the full covariance matrix. The cell e.s.d.'s are taken into account individually in the estimation of e.s.d.'s in distances, angles and torsion angles; correlations between e.s.d.'s in cell parameters are only used when they are defined by crystal symmetry. An approximate (isotropic) treatment of cell e.s.d.'s is used for estimating e.s.d.'s involving l.s. planes.
Refinement. Refinement of *F*^2^ against ALL reflections. The weighted *R*-factor *wR* and goodness of fit *S* are based on *F*^2^, conventional *R*-factors *R* are based on *F*, with *F* set to zero for negative *F*^2^. The threshold expression of *F*^2^ > σ(*F*^2^) is used only for calculating *R*-factors(gt) *etc*. and is not relevant to the choice of reflections for refinement. *R*-factors based on *F*^2^ are statistically about twice as large as those based on *F*, and *R*- factors based on ALL data will be even larger.

### Fractional atomic coordinates and isotropic or equivalent isotropic displacement parameters (Å^2^)

**Table:**

	*x*	*y*	*z*	*U*_iso_*/*U*_eq_	
Cu1	0.85578 (2)	0.959759 (8)	0.015468 (13)	0.03310 (6)	
C1	0.7588 (2)	1.14438 (8)	0.17923 (12)	0.0428 (3)	
C2	0.5838 (2)	1.13393 (8)	0.22195 (12)	0.0449 (3)	
H2	0.5207	1.0935	0.2093	0.054*	
C3	0.5037 (3)	1.18455 (9)	0.28383 (12)	0.0484 (4)	
C4	0.5971 (3)	1.24540 (9)	0.30041 (16)	0.0588 (5)	
H4	0.5442	1.2790	0.3426	0.071*	
C5	0.7672 (3)	1.25604 (9)	0.25470 (16)	0.0626 (5)	
H5	0.8271	1.2975	0.2642	0.075*	
C6	0.8508 (3)	1.20571 (9)	0.19449 (15)	0.0547 (4)	
H6	0.9671	1.2129	0.1646	0.066*	
C7	0.8464 (2)	1.08987 (8)	0.11286 (11)	0.0401 (3)	
C8	0.2380 (3)	1.11606 (12)	0.32315 (18)	0.0671 (5)	
H8A	0.2120	1.1065	0.2500	0.101*	
H8B	0.1237	1.1189	0.3619	0.101*	
H8C	0.3131	1.0803	0.3521	0.101*	
C9	0.7489 (2)	1.07841 (8)	−0.26044 (12)	0.0428 (3)	
C10	0.5683 (2)	1.05961 (9)	−0.28718 (12)	0.0461 (4)	
H10	0.5042	1.0283	−0.2458	0.055*	
C11	0.4845 (3)	1.08807 (10)	−0.37628 (14)	0.0555 (4)	
C12	0.5780 (3)	1.13632 (12)	−0.43556 (16)	0.0695 (6)	
H12	0.5201	1.1568	−0.4931	0.083*	
C13	0.7561 (4)	1.15386 (13)	−0.40930 (17)	0.0787 (7)	
H13	0.8192	1.1856	−0.4503	0.094*	
C14	0.8439 (3)	1.12486 (10)	−0.32223 (15)	0.0614 (5)	
H14	0.9655	1.1366	−0.3057	0.074*	
C15	0.8413 (2)	1.04876 (7)	−0.16352 (11)	0.0389 (3)	
C16	0.2271 (3)	1.01287 (14)	−0.3747 (2)	0.0789 (7)	
H16A	0.3109	0.9755	−0.3852	0.118*	
H16B	0.1147	1.0047	−0.4135	0.118*	
H16C	0.1991	1.0172	−0.3007	0.118*	
C17	0.5228 (3)	0.84701 (10)	0.04591 (16)	0.0570 (4)	
C18	0.3797 (3)	0.79457 (13)	0.0586 (2)	0.0804 (7)	
H18A	0.3836	0.7770	0.1295	0.121*	
H18B	0.4019	0.7583	0.0092	0.121*	
H18C	0.2599	0.8141	0.0452	0.121*	
N1	0.6281 (2)	0.88878 (8)	0.03641 (12)	0.0559 (4)	
O1	0.75919 (17)	1.03482 (5)	0.10169 (10)	0.0476 (3)	
O2	1.00189 (17)	1.10313 (6)	0.07340 (10)	0.0503 (3)	
O3	0.33498 (19)	1.17917 (7)	0.33120 (11)	0.0611 (3)	
O4	0.75562 (16)	1.00329 (6)	−0.11367 (9)	0.0458 (3)	
O5	0.99825 (17)	1.07201 (6)	−0.13874 (9)	0.0492 (3)	
O6	0.3093 (2)	1.07285 (10)	−0.41088 (13)	0.0801 (5)	

### Atomic displacement parameters (Å^2^)

**Table:**

	*U*^11^	*U*^22^	*U*^33^	*U*^12^	*U*^13^	*U*^23^
Cu1	0.03442 (10)	0.03078 (9)	0.03410 (10)	−0.00225 (6)	−0.00097 (6)	0.00015 (6)
C1	0.0519 (9)	0.0379 (7)	0.0386 (7)	0.0095 (6)	−0.0042 (6)	−0.0037 (6)
C2	0.0551 (10)	0.0401 (7)	0.0394 (7)	0.0106 (7)	−0.0036 (7)	−0.0036 (6)
C3	0.0580 (10)	0.0463 (8)	0.0410 (8)	0.0182 (7)	−0.0024 (7)	−0.0031 (6)
C4	0.0748 (13)	0.0453 (9)	0.0561 (10)	0.0156 (9)	−0.0011 (9)	−0.0123 (7)
C5	0.0797 (14)	0.0404 (9)	0.0677 (12)	0.0021 (9)	−0.0052 (10)	−0.0142 (8)
C6	0.0595 (11)	0.0454 (9)	0.0593 (10)	0.0022 (8)	−0.0016 (8)	−0.0082 (7)
C7	0.0459 (8)	0.0380 (7)	0.0365 (7)	0.0056 (6)	−0.0040 (6)	−0.0017 (5)
C8	0.0561 (12)	0.0765 (14)	0.0687 (12)	0.0080 (10)	0.0022 (9)	−0.0186 (10)
C9	0.0523 (9)	0.0392 (7)	0.0367 (7)	0.0038 (6)	−0.0064 (6)	0.0014 (6)
C10	0.0530 (10)	0.0461 (8)	0.0390 (7)	0.0048 (7)	−0.0053 (7)	0.0040 (6)
C11	0.0599 (11)	0.0621 (11)	0.0444 (8)	0.0071 (8)	−0.0117 (8)	0.0039 (7)
C12	0.0854 (16)	0.0740 (13)	0.0492 (10)	−0.0003 (11)	−0.0182 (10)	0.0206 (9)
C13	0.0931 (17)	0.0831 (15)	0.0598 (12)	−0.0198 (13)	−0.0127 (11)	0.0347 (11)
C14	0.0682 (13)	0.0629 (11)	0.0531 (10)	−0.0130 (9)	−0.0114 (9)	0.0144 (8)
C15	0.0466 (9)	0.0356 (7)	0.0347 (7)	0.0044 (6)	−0.0036 (6)	−0.0012 (5)
C16	0.0613 (14)	0.1005 (19)	0.0749 (15)	−0.0078 (13)	−0.0137 (11)	0.0090 (13)
C17	0.0519 (10)	0.0576 (10)	0.0615 (11)	−0.0063 (8)	−0.0024 (8)	0.0131 (8)
C18	0.0665 (14)	0.0763 (15)	0.0984 (17)	−0.0306 (11)	−0.0125 (12)	0.0349 (13)
N1	0.0525 (9)	0.0597 (9)	0.0553 (9)	−0.0205 (7)	−0.0018 (7)	0.0092 (7)
O1	0.0501 (7)	0.0416 (6)	0.0511 (6)	0.0006 (5)	0.0081 (5)	−0.0104 (5)
O2	0.0510 (7)	0.0418 (6)	0.0581 (7)	−0.0008 (5)	0.0076 (5)	−0.0112 (5)
O3	0.0631 (8)	0.0580 (8)	0.0623 (8)	0.0173 (6)	0.0086 (6)	−0.0118 (6)
O4	0.0477 (6)	0.0489 (6)	0.0408 (5)	−0.0043 (5)	−0.0083 (5)	0.0093 (5)
O5	0.0511 (7)	0.0499 (6)	0.0465 (6)	−0.0077 (5)	−0.0123 (5)	0.0122 (5)
O6	0.0659 (10)	0.1017 (12)	0.0726 (10)	−0.0032 (9)	−0.0282 (8)	0.0271 (9)

### Geometric parameters (Å, °)

**Table:**

Cu1—O2^i^	1.9591 (11)	C9—C10	1.395 (2)
Cu1—O1	1.9606 (11)	C9—C15	1.510 (2)
Cu1—O4	1.9769 (11)	C10—C11	1.394 (2)
Cu1—O5^i^	1.9791 (11)	C10—H10	0.9300
Cu1—N1	2.1703 (14)	C11—O6	1.369 (2)
Cu1—Cu1^i^	2.6416 (3)	C11—C12	1.383 (3)
C1—C2	1.388 (2)	C12—C13	1.370 (3)
C1—C6	1.389 (2)	C12—H12	0.9300
C1—C7	1.499 (2)	C13—C14	1.390 (3)
C2—C3	1.390 (2)	C13—H13	0.9300
C2—H2	0.9300	C14—H14	0.9300
C3—O3	1.360 (2)	C15—O4	1.2554 (19)
C3—C4	1.388 (3)	C15—O5	1.2594 (19)
C4—C5	1.371 (3)	C16—O6	1.396 (3)
C4—H4	0.9300	C16—H16A	0.9600
C5—C6	1.385 (3)	C16—H16B	0.9600
C5—H5	0.9300	C16—H16C	0.9600
C6—H6	0.9300	C17—N1	1.124 (2)
C7—O2	1.254 (2)	C17—C18	1.467 (3)
C7—O1	1.2588 (19)	C18—H18A	0.9600
C8—O3	1.427 (3)	C18—H18B	0.9600
C8—H8A	0.9600	C18—H18C	0.9600
C8—H8B	0.9600	O2—Cu1^i^	1.9591 (11)
C8—H8C	0.9600	O5—Cu1^i^	1.9791 (11)
C9—C14	1.382 (3)		
			
O2^i^—Cu1—O1	168.03 (5)	C14—C9—C10	120.08 (15)
O2^i^—Cu1—O4	89.55 (5)	C14—C9—C15	119.54 (15)
O1—Cu1—O4	90.13 (5)	C10—C9—C15	120.38 (14)
O2^i^—Cu1—O5^i^	88.43 (6)	C11—C10—C9	119.58 (17)
O1—Cu1—O5^i^	89.42 (5)	C11—C10—H10	120.2
O4—Cu1—O5^i^	168.11 (5)	C9—C10—H10	120.2
O2^i^—Cu1—N1	93.49 (6)	O6—C11—C12	115.27 (16)
O1—Cu1—N1	98.44 (6)	O6—C11—C10	124.71 (18)
O4—Cu1—N1	95.86 (5)	C12—C11—C10	120.00 (18)
O5^i^—Cu1—N1	95.96 (5)	C13—C12—C11	119.90 (17)
O2^i^—Cu1—Cu1^i^	83.13 (4)	C13—C12—H12	120.0
O1—Cu1—Cu1^i^	84.93 (4)	C11—C12—H12	120.0
O4—Cu1—Cu1^i^	84.64 (3)	C12—C13—C14	120.98 (19)
O5^i^—Cu1—Cu1^i^	83.48 (4)	C12—C13—H13	119.5
N1—Cu1—Cu1^i^	176.58 (5)	C14—C13—H13	119.5
C2—C1—C6	120.59 (15)	C9—C14—C13	119.39 (19)
C2—C1—C7	119.64 (14)	C9—C14—H14	120.3
C6—C1—C7	119.74 (16)	C13—C14—H14	120.3
C1—C2—C3	119.36 (16)	O4—C15—O5	125.17 (14)
C1—C2—H2	120.3	O4—C15—C9	117.60 (14)
C3—C2—H2	120.3	O5—C15—C9	117.23 (13)
O3—C3—C4	115.76 (15)	O6—C16—H16A	109.5
O3—C3—C2	124.28 (17)	O6—C16—H16B	109.5
C4—C3—C2	119.96 (18)	H16A—C16—H16B	109.5
C5—C4—C3	120.11 (16)	O6—C16—H16C	109.5
C5—C4—H4	119.9	H16A—C16—H16C	109.5
C3—C4—H4	119.9	H16B—C16—H16C	109.5
C4—C5—C6	120.75 (18)	N1—C17—C18	177.7 (2)
C4—C5—H5	119.6	C17—C18—H18A	109.5
C6—C5—H5	119.6	C17—C18—H18B	109.5
C5—C6—C1	119.18 (19)	H18A—C18—H18B	109.5
C5—C6—H6	120.4	C17—C18—H18C	109.5
C1—C6—H6	120.4	H18A—C18—H18C	109.5
O2—C7—O1	125.48 (14)	H18B—C18—H18C	109.5
O2—C7—C1	116.75 (14)	C17—N1—Cu1	173.08 (18)
O1—C7—C1	117.77 (15)	C7—O1—Cu1	122.06 (11)
O3—C8—H8A	109.5	C7—O2—Cu1^i^	124.38 (10)
O3—C8—H8B	109.5	C3—O3—C8	118.32 (14)
H8A—C8—H8B	109.5	C15—O4—Cu1	122.76 (10)
O3—C8—H8C	109.5	C15—O5—Cu1^i^	123.93 (10)
H8A—C8—H8C	109.5	C11—O6—C16	118.17 (16)
H8B—C8—H8C	109.5		

Symmetry codes: (i) −*x*+2, −*y*+2, −*z*.
